# Sonic hedgehog signaling in epithelial tissue development

**DOI:** 10.1051/rmr/190004

**Published:** 2019-12-31

**Authors:** Lu Zheng, Chen Rui, Hao Zhang, Jing Chen, Xiuzhi Jia, Ying Xiao

**Affiliations:** Central Lab of Biomedical Research Center, Sir Run Run Shaw Hospital, School of Medicine, Zhejiang University Hangzhou PR China

**Keywords:** Sonic hedgehog, epidermis, skin appendages, mammary gland, gastric epithelium, intestinal epithelium

## Abstract

The Sonic hedgehog (SHH) signaling pathway is essential for embryonic development and tissue regeneration. The dysfunction of SHH pathway is involved in a variety of diseases, including cancer, birth defects, and other diseases. Here we reviewed recent studies on main molecules involved in the SHH signaling pathway, specifically focused on their function in epithelial tissue and appendages development, including epidermis, touch dome, hair, sebaceous gland, mammary gland, tooth, nail, gastric epithelium, and intestinal epithelium. The advance in understanding the SHH signaling pathway will give us more clues to the mechanisms of tissue repair and regeneration, as well as the development of new treatment for diseases related to dysregulation of SHH signaling pathway.

## 1 Introduction

### 1.1 SHH signaling pathway

In the development of invertebrates and vertebrates, the members of the hedgehog (HH) signaling family mediate many essential processes, including short-term and long-term modeling. The *Hh* gene family encodes a secreted protein that plays a critical regulatory role in the process of embryogenesis and environmental stabilization in adult tissues of invertebrates and vertebrates [44]. In the fly, signals are regulated by a single *Hh* gene, while in mammals, Sonic (SHH), Indian (IHH), and Desert (DHH) hedgehog play different regulatory roles [37,148].

The SHH pathway should be off at most of the time, by the inhibition of specific factors such as Patched 1 (PTC1), Protein Kinase A (PKA), Casein Kinase1 (CK1), and Glycogen Synthase Kinase 3 beta (GSK3b), and only valid at precise time points [98]. It plays a biological role by terminating the factor of glioma-associated oncogene (*Gli*) [144]. In this way, SHH mediates various reactions by the regulation between activator form and repressor form. IHH is a core factor in the morphogenesis of vertebrate skeleton. IHH signal induces activation of Parathyroid Hormone-related Protein (PTHRP) on the articular surface and gets rid of the hypertrophy of chondrocytes [49]. It also participates in endochondral bone formation as a negative regulator of chondrocyte differentiation in mouse [131] and is involved in the development of gastrointestinal tract [127] and mammary glands [56]. Meanwhile, DHH plays a vital role in HH signaling. DHH/PTC1 signaling triggers differentiation of Leydig cells by up-regulating Steroidogenic Factor 1 and expression of cytochrome P450 Side-Chain Cleavage enzyme located outside testicular cord [145].

HH ligands are tissue-specific expressed, but may also be co-expressed in the same tissue. For example, *Shh*, *Dhh*, and *Ihh* are specifically expressed in the adrenal, testis, and endochondral bones, respectively [61], whereas both of SHH and IHH are detected in organs such as embryonic heart [148], gut [11], bladder [39], prostate [59] and stomach [89]. The interactions of the three HH factors are complicated. In the adrenal gland, SHH and DHH act synergistically [114]; In the *Dhh*-mutant epididymis, SHH plays a compensatory role in order to maintain the stability of the signaling pathway [21].

### 1.2 Molecules and their functions in the SHH signaling pathway

**Fig. 1:**
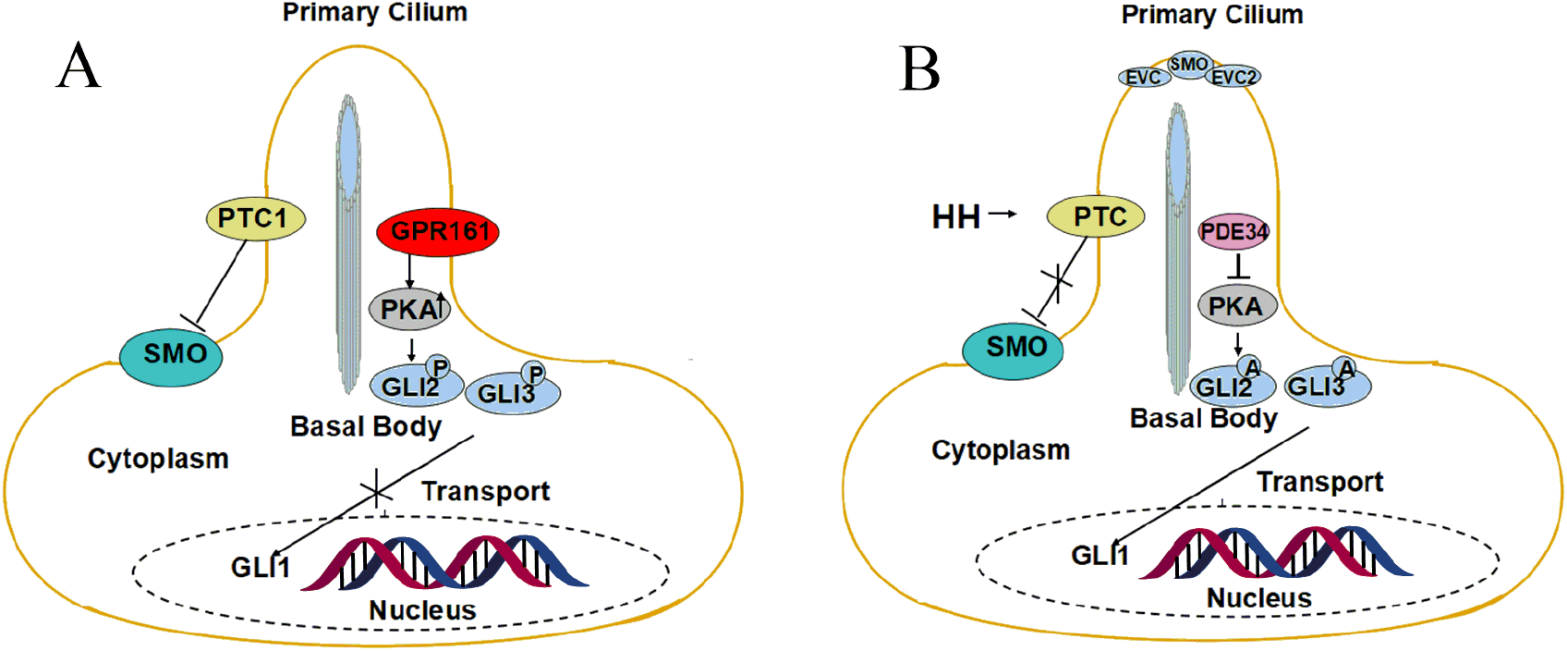
HH signaling pathway in the primary cilium. In the absence of SHH, PTC1 is located at PC, indirectly inhibiting accumulation of SMO by transporting putative sterol ligand outside with a ‘tunnel’ structure. Meanwhile, PKA is up-regulated by GPR161, located at the base of PC, through increasing cAMP level. GLI2 and GLI3 are then phosphorylated by PKA, CK1, GSK3b, and sequestered by SUFU. Phosphorylated GLI2 and GLI3 are easy to get cleaved and repress the GLI-dependent transcription of target genes. In the presence of SHH ligand, the inhibit of SMO declined by the interaction between SHH and PTC1. SMO forms a complex with EVC and EVC2, and transports into PC structure. Level of cAMP is then down-regulated, preventing GLI2 and GLI3 from being phosphorylated. Full-length GLI2 and GLI3 actives GLI1 to translocate to the nucleus and induces the expression of HH target genes.

The conduction of SHH signaling pathway requires the participation of many proteins and factors (Tab. 1), mainly including SHH, PTC receptors (PTC1, PTC2), Smoothened receptor (SMO), Kinesin Family member7 (KIF7), Suppressor of fused homolog (SUFU), PKA, and GLI transcription factors (GLI1, GLI2, GLI3) [130] (Fig. 1). In the absence of SHH, PTC and G Protein-coupled Receptor161 (GPR161) are located at the base of primary cilium (PC), while accumulation of SMO is diminished by transporting sterol-like SMO ligand outside by a ‘tunnel’ structure of PTC1. GLI2 and GLI3 are phosphorylated by PKA, CK1, and GSK3b, which are sequestered by SUFU. Herein, GPR161 acts as an activator of PKA, which is up-regulated by Adenosine Cyclophosphate (cAMP) in PC structure. Phosphorylated GLI2 and GLI3 are processed into repressor forms by the proteasome and repress the GLI-dependent transcription of target genes. In the presence of SHH, PTC1 and GPR161 are transferred out of PC upon binding with SHH, declining the inhibition of SMO, and activating its movement into PC. SMO forms a sophisticated structure with EVC and EVC2 to proceed signal transduction of HH pathway. Activated SMO reduces the suppression of GLI2 and GLI3 mediated by SUFU. The exit of GPR161 from PC weakens the level of cAMP and PKA, which can help GLI2 and GLI3 to maintain their full-length forms without being phosphorylated and active GLI1. Finally, the activated form of GLI1 translocates into the nucleus and induces the expression of HH target genes [141].

**Table 1:** HH pathway modulators.

Factor effected	Functions	Modulators	References (PMID)
HH	Antagonist	RU-SKI 39 RU-SKI41 RU-SKI43 RU-SKI 50	[81]
SHH	Block	5E1	[26]
PC	Antagonist	CA1 CA2	[142]
SMO	Antagonist	DHCEO SMANT KAAD-cyclopamine, Jervine Saridegib Vitamin D_3_ SEN450	[101] [137] [113] [12] [123] [10] [29]
SMO	Agonist	SAG Purmorphamine, Oxysterols Cholesterol, PI4P GSA-10	[30] [105] [69] [63] [47] [33]
GLI	Antagonist	Arsenic trioxide	[7]

#### 1.2.1 PC

Cilia are microtubule organelles that exist in most eukaryotic cells, and they can be classified as inactive (Primary Cilia, PC) or active cilia. During SHH signaling in vertebrates, the primary cilium acts as a signaling antenna by preferentially localizing selected components [76]. Nearly all kinds of interphase vertebrate cells possess a primary cilium structure that extends into the extracellular environment to work as mechanical models and mechanical sensors [22], which is also crucial in determining the left-right axis asymmetry of the process development [85]. Although the motile cilia produce extracellular fluid flow or drive a single cell, the primary cilia have long been considered to be remaining [8]. The cilia and flagella contain the axoneme with 9 peripheral microtubule cores. In the active cilia, these diploids are surrounded by a pair of central microtubules, which can be called “9+2” superstructure while the lack of central pair can make the primary cilium axoneme as “9+0” arrangement [23].

The primary cilium is essential for regular anterior segment (AS) development during eye embryogenesis, and primary cilium defects may lead to diseases like congenital glaucoma. Recent evidence suggests a unique structure composed of lipid membrane, a key role for cilia function and HH signaling, demonstrating a link between cilia regulation of phosphoinositides by inositol polyphosphate 5-phosphatase (INPP5E) and SHH regulation via ciliary trafficking of Tubby-like protein 3 (TULP3)/GPR161, and also provide mechanistic vision into ciliary alterations found in Serge N. Schiffmann syndromes resulting from INPP5E mutations [17]. Ras genes of rat brain-35 (RAB35) regulate the transport pathways necessary for the formation, function, and composition of primary cilia in mammalian cells and zebrafish.

#### 1.2.2 SHH

A vertebrate gene, which is homologous to the Drosophila segmental polarity gene, was cloned and named *Shh* in 1993. SHH is a protein secreted from the zone of polarizing activity (ZPA) with a tetrahedrally coordinated Zn^2+^ cation and Ca^2+^ cation, and acts as a morphogenetic factor, spreading in a gradient from back to front in the whole limb field. It acts as a ligand for membrane-bound receptors (such as PTC) rather than as an active protease [31]. SHH is a 45 kDa precursor protein, and full-length SHH protein has no activity in binding with PTC1 [100]. During the biosynthetic process, SHH precursors are autocatalyzed on the endoplasmic reticulum to release an amino-terminal signal domain (SHH-N), whose carboxyl-terminal is covalently bound to cholesterol. SHH acyltransferase then adds palmitate α-amino group of amino specific cysteine to produce mature double-lipidated signal molecule [83]. It has already known that cholesterol is a lipophilic part, covalently connected to the amino-terminal signaling domain during its processing, with the carboxyl-terminal as an intramolecular structure [84]. The N- and C-modifications are necessary for producing founctional SHH, the mechanism can be described as follows : (1) SHH-N with aliphatic acylation is more active than unacylated SHH-N, as determined by differentiation analysis and HH signaling analysis; (2) blocking HH-N palmitoglycation (by mutation of its palmitoglycation site) affects embryonic development in Mice and Drosophila; HH acyltransferase inhibitors prevent SHH from getting palmitoylated also blocks HH signaling.

The other fragment is 25 kDa C-terminal fragment with automatic processing mechanism activity [35,97]. Traci M. Tanaka Hall et al. identified a 17 kDa fragment of HH-C (HH-C17) that plays a role in initiating automatic processing and reported its crystal structure, leading to intramolecular cleavage of the full-length HH protein and partial covalent attachment of cholesterol to the newly formed amino-terminal fragment [35].

Fan jiajun et al. have reported that vismodegib, an inhibitor of SHH pathway, had only a slight anti-tumor effect on A549 and NCI-H1975 lung adenocarcinoma cells, with *Gli2* overexpressed and autophagy activity increased [27]. The deformities associated with *Shh* gene mutations highlight the importance of SHH in embryonic development [95]. *Shh* knockout mice have developmental defects that eventually lead to their embryonal lethal phenotype.

#### 1.2.3 PTC

All initiation signaling of the SHH pathway is started by binding to its receptor, PTC receptors (PTC1 and PTC2) [96]. Both PTC receptors have been shown to exhibit HH ligand-binding activity with similar affinity and form a complex with SMO, while concomitant loss of PTC1 and PTC2 activity inhibits epidermal differentiation.

Compared with PTC2, the function of the mammalian PTC1 paralogue is quite clear. Human PTC1 consists of 1,447 amino acids, including three approximately 30-kD soluble domains, 12 transmembrane helices, and two extracellular domains (ECD-I and ECD-II), whose function is to bind HH-N and one cytoplasmic carboxyl-terminal domain (CTD). Besides, transmembrane helices 2-6 (TM2-TM6) of PTC are predicted to form a sterol-sensing domain (SSD), which is involved in cholesterol metabolism and signaling in other proteins such as Niemann-Pick C1 (NPC1) and 3-hydroxy-3-methylglutaryl coenzyme-A (HMG-CoA) reductase [32]. Xiaofeng Qi et al. discovers that the structure of PTC1 has internal two-fold pseudo-symmetry in the transmembrane core, which features two homologous extracellular domains and a sterol-sensing domain [86].

There are 3 endogenous sterol-like densities in the PTC1 protein: the first in the extracellular domain I, the second in the sterol-sensing domain, and the last in the N terminus of TM [87]. The interactions between SHH and PTC was confirmed by R. Blake Pepinsky, who constructed mutants of 11 selected amino acid residues around the surface of SHH into cysteine residues using mapping strategy. Regardless of the attachment size, about one-third of the SHH surface can be modified without affecting the function, and Asn-50 and Ser-156 sites are very close to PTC binding sites [79].

Unliganded PTC inhibits SHH signaling, and this repression could be released when HH binds to PTC. Early in 1998, Chen et al. ensured that PTC usually binds to SHH without the help of SMO [18]. Specifically, after HH binding, PTC releases its repression of SMO. A recent structure study suggests that HH binding may be close to an inside ‘tunnel’ structure in PTC1, which transport sterol ligand to accumulate on the membrane for SMO, and then SMO activates GLI proteins, which transcripts HH target genes that promote cell proliferation and differentiation activation [86].

The PTC1 ciliary intensity is regulated by truncating PTC cytoplasmic tail length, leading to highly correlated SMO inhibition, which strongly indicates the critical role of ciliary PTC1 in inhibiting SMO activity [50]. Inhibition of PTC1 in vertebrates is caused by direct binding with N-terminal of palmitoylated SHH, which is sufficient enough to inhibit PTC1 and activate signal transduction [124].

#### 1.2.4 HHIP

HHIP is a surface receptor antagonist that is equally effective against all three mammalian HH homologs, which was identified and named in 1999 by Chuang and McMahon [21]. Computational analysis of the HHIP ECD sequence suggested the presence of four distinct globular domains: a cysteine-rich N-terminal domain with a Frizzled (Fz) fold, a central six-bladed β-propeller, and two C-terminal epidermal growth factor (EGF) repeats. HHIP competes for SHH binding to PTC1 and has a stronger affinity than PTC1. HHIP and PTC1 both bind at the SHH pseudo active site groove with Zn^2+^ cation competitively. The study of HHIP- and PTC1-peptide binding suggest a ‘patch for Patched’ at the SHH pseudo active site [13].

#### 1.2.5 GPR161

GPR161 is a PKA substrate and also has an A-Kinase Anchoring Protein (AKAP) motif embedded in its C-terminal tail and creates a cAMP-sensing signalosome. In addition, GPR161 is effective in recruiting isoform-specific PKA complexes to primary cilia. Mukhopadhyay, et al. identify the cilium-localized orphan GPR161 to be a negative regulator of SHH signaling during development of neural tube [68]. *Gpr161* is a tumor suppressor gene of SHH subtype medulloblastoma (MB). The deletion of GPR161 increases the downstream activity of SHH pathway by downregulating GLI3-mediated inhibition. It is further found that early deletion of *Gpr161* during embryogenesis increases the incidence and severity of tumors [103].

#### 1.2.6 SMO

SMO protein is a key signal transducer in the HH signaling pathway, responsible for maintaining normal embryonic development and participating in carcinogenesis [133].

SMO belongs to the F-class of the G-protein-coupled receptor (GPCR) superfamily and is characterized by extracellular cysteine-rich domain (CRD), followed by extracellular linker domain (LD) that connect the 7-TM domain. Similar to the classical GPCRs, it has been proposed that the output of SMO is controlled by a balance of active and inactive conformations, regulated by small molecular ligands [112]. Recently, cholesterol is identified as the endogenous SMO agonist [43]. Nachtergaele et al. present the 2.3 Å crystal structure of vertebrate SMO with the extracellular cysteine-rich domain [70]. CRD is a crucial regulatory module of SMO activation and is necessary to respond to the signal transduction of natural HH ligand. The secreted amino-terminal domain seems to be responsible for signal transduction activity, while the carboxyl-terminal domain contains self-processing activity. It has been reported that the domain may act an essential role in hydrolase by analyzing the crystal structure of SHH amino-terminal domain [36]. Previous structural studies have shown that SMO CRD can bind to sterol-like ligands to regulate SMO activity [63]. SMO contains a ligand-binding pocket in its 7-TM region that can bind ligands and act as agonist or antagonist [134]. Qi et al. demonstrate that 24,25-epoxycholesterol can function as an endogenous ligand of PTC1, which can stimulate HH signaling in cells and can trigger G-protein signaling via human SMO *in vitro* [88].

#### 1.2.7 SUFU

In the absence of SHH, SUFU makes the regulation of truncating GLI2 and GLI3. Meanwhile, it releases full-length GLI in the cytoplasm, protecting a pool of potential proteins that can be converted to be activators or repressors as needed. This character makes SUFU at the center factor of the pathway and a key regulator of all GLI factors. Activation of the pathway promotes the dissociation of the SUFU-GLI complex and the nuclear translocation of GLI protein [117,149]. SUFU also interacts with GLI2 in the nucleus to inhibit GLI2 activity [38], co-occupies HH target gene promoter with GLI protein, p66β, a chromatin-remodeling factor, and myelocytomatosis binding protein (MYCBP), a transcriptional regulatory factor [38].

#### 1.2.8 KIF7

KIF7 is a mammal homologous to Drosophila Costal2 (Cos2), has the function of regulating signal transmission from SMO to GLI. The accumulation of KIF7 in the primary cilia is regulated by HH [25]. Human KIF contains an N-terminal kinematic domain, consisted of a nucleotide-binding region an microtubule (MT) interaction region, followed by a responsible oligomeric helix and a C-terminal globular tail domain [52]. Without stimulation, KIF7 locates at the base of primary cilia, thus prevent GLI2 and GLI3 accumulation. KIF7 mutant is effective to deregulation of GLI transcription process of target genes [58].

#### 1.2.9 GLI

The mammalian counterpart of Drosophila Ci is GLI transcription factors. GLI factors are zinc finger Glioma-associated transcription factors, which mediate the transcription of HH target genes. There are three GLI homologies in mammals, GLI2 and GLI3 are bifunctional transcription factors with both activation (C-terminal) and inhibition (N- terminal) domains, which can be both as activators or repressors in the signaling pathway. Due to the lack of N-terminal inhibitory domain, GLI1 can only act as a transcriptional activator, regulated by phosphorylation/maintenance of the full length of GLI2 and GLI3 [45].

The activation of GLI transcription factors in the nucleus promotes the transcription of various target genes, including those involved in SHH pathway feedback, such as PTC1, proliferation promoting genes, cell cycle regulators, and apoptosis regulators [73]. The results of this pathway depend on the balance between the activation and inhibition of GLI protein. Research in 2019 has found that the maximum activation of GLI1 is dependent on cilia, but in the absence of cilia, there is partial activation of GLI1 by HH signal mediated by SMO [136].

In the absence of HH ligands, GLI3 is the central inhibitor of this pathway, while in their presence, GLI2 is the main HH effector for *Gli1* expression [15]. SHH inhibits the proteolysis of full-length GLI3, remains the untruncated form of GLI3 and promotes the activity of full-length GLI1, 2 and 3 activators [60,132]. Recent studies have found that SHH/GLI2 and SHH/GLI3 signals are necessary for the healthy development of mouse placentas, and may be important factors for pregnant maintenance [77].

#### 1.2.10 Other molecules

The regulation of PTC1 on HH signal on cell surface is finely controlled by many other cell surface molecules, such as the invertebrate proteins interference hedgehog (IHOG) [64], brother of IHOG (BOI) [146], their corresponding vertebrate homologs (CDON) [147] and brother of CDON (BOC) [119]. Although CDON and IHOG are similar in function, IHOG binds to a different surface near the second HH helix, and this interaction requires heparin, which not only connects two binding partners, but also promotes IHOG dimerization [67], whereas CDON and BOC can each directly bind to SHH.

#### 1.2.11 Non-canonical signaling pathway

In addition to the classical typical HH-PTC-SMO pathway, there is increasing evidence that non-canonical signaling pathways for GLI activation also play significant roles. Non-canonical pathway refers to independent response of all cells and tissues to any factors in HH signaling pathway, and gets transcriptional changes mediated by the GLI family [14]. In some cancer type, canonical and non-canonical activation may co-exist [82].

The non-canonical pathway GLI-independent mechanisms can be identified of two types: Type I is about modulation of Ca^2+^ and actin cytoskeleton, which is downstream of SMO; while type II is signaling pathway that is independent on SMO [94].

There are kinds of non-canonical pathways that positively promote HH signal transduction, including RAS-RAF-MEK-ERK signaling pathway [92], PI3K-AKT-mTOR signaling pathway [104], TGFβ signaling pathway [24]; and pathways that inhibit HH signaling such as MAPKKK-MEKK [4].

## 2 SHH pathway functions in tissue development

Previous study of experimental embryology shows that the organ development of embryo is mainly regulated by the so-called induced tissue interactions, which mostly occurs between epithelial tissues and mesenchymal tissues. SHH signaling pathway plays a crucial role in epithelial tissue development.

### 2.1 Skin and its appendages

#### 2.1.1 Epidermis

In the vertebrate kingdom, epithelial appendages begin to form during embryogenesis, when progenitor cells within WNT signaling epithelium are lined up into morphologically similar substrates. After the formation of similar epidermal buds, the selection of appendages depends on the antagonism between bone morphogenetic proteins (BMPs) and SHH signals in different skin regions in the mesenchyme. When the SHH signal is active, it determines the formation of hair follicles (HFs). When SHH signal is weaker than BMP, it determines the formation of sweat glands [62]. SHH signals from epithelial mesenchyme affect the fate of skin appendages.

#### 2.1.2 Touch dome

In the embryo, SHH signals from the epithelium also stimulate the formation of Gli1^+^ cells and lead to an increase in *S*ox9^+^ cells, which develop into hair follicle stem cells (HFSCs) and hair follicle line cells. The researchers further confirm that Sox9-mediated fibroblast growth factor receptor2 (FGFR2) signals increase the production of Merkel cells (MCs), a tactile receptor, which is located in the touch dome (TD) [71]. The touch dome expresses keratin 17, and Merkel cells are located in it and adjacent to keratin 15 expressing keratinocytes.

When the placode begins to develop, SHH signals from the downstream of primary hair follicle development pathway Ectodysplasin A/Ectodysplasin A receptor (EDA/EDAR) affects the formation of TD MCs [143]. Other researchers have confirmed that loss of Polycomb Repressive Complex 2 (PRC2) in the epidermis results in the formation of ectopic Merkel cells. By upregulation of known PRC2 target genes, PRC2 deletion extends the range of differentiated epidermal cells to Merkel cells. Importantly, PRC2-mediated inhibition of the Merkel cell differentiation program requires the induction of SHH signals to form mature Merkel cells [80].

SHH signal of self-neurons also plays a crucial role in maintaining the development of touch dome. Skin denervation experiment suggests that the regeneration of touch dome stem cells needs a neural microenvironment. The removal of SHH from neurons or epidermal SMO indicates that SHH is a necessary niche factor for maintaining touch dome stem cells. Up-regulation of the HH signaling pathway results in tumor amplification of touch dome keratinocytes, but not neoplasia of Merkel cells. Neurogenic SHH is a key regulator of lineage-specific stem cells, maintaining the special sensory compartments of the epidermis. In conclusion, SHH is necessary for the development of touch dome.

#### 2.1.3 Hair

Hair follicle development can be divided into three stages: induction, organogenesis and cytodifferentiation [78]. HH signal transduction is crucial for both the transition from induction stage to organogenesis stage, and the downward growth of epithelial cells after induction stage. Mice lacking SHH produce hair placodes and dermal condensates, but the formation of hair follicles is blocked at the hairy bud stage [19,109]. At the beginning of the organogenesis stage in E15.5, PTC1, SMO, GLI1 and GLI2 can be detected in the dermal components of early hair follicles, indicating that HH signal also participate in regulation of dermal cells. SMO inhibition in hair cells mediated by RNAi results in the loss of the dermal papilla (DP) precursor and hair cell condensate, bringing out the result of cease growing during organogenesis, similar to similar to *SHH^−/−^* skin. These results indicate that the dermal SHH signaling modulates specific DP signals to maintain DP maturity [140]. SHH signaling is not needed to start hair follicle development, but it is quite important to control hair follicle growth and morphogenesis.

In addition, the stem cells in the hair follicle bulge can regenerate hair follicles periodically, while the stem cell population maintaining the epidermis is unique. During telogen, GLI1 expression is limited to two different epidermal stem cell domains [16], Gli ^+^/Lgr5^+^ stem cells in contact with hair germ and Gli ^+^/Sox9^+^ stem cells in the upper fringe of the bulge [6,135]. Gli ^+^/Lgr5^+^ stem cells are regulated by SHH signaling pathway in DP and differentiate into new hair follicle structures in the hair follicle growth cycle. The upper bulge that expresses GLI1 is induced by the release of SHH from nerve cells associated with bulge. Therefore, upper bulge cells may constitute a long-term stem cell bank, ready to contribute to the repair of the epidermis between follicles.

#### 2.1.4 Sebaceous gland

The mature sebaceous glands (SGs) are the appendages of the total secretion of epidermal acini. As an important part of the pilosebaceous gland, SG is connected with the junction zone (JZ), which is located in the upper permanent part of HF. SG is the last lineage to develop at E16.5-E17.5 (bulbous peg stage) in the morphogenesis of HF, which keeps a permanent relationship with the upper part of HF. HH signaling pathway is a typical signaling pathway involved in the proliferation of sebocytes. The expression of activated SMO in mouse epidermis leads to the formation of ectopic SGs. On the contrary, inhibition of HH signal by overexpression of dominant-negative GLI mutant can inhibit the differentiation of sebocytes [2]. The inhibition of HH pathway selectively inhibited the development of sebocytes, and the activation of HH pathway resulted in a significant increase in the size and number of sebaceous glands. In conclusion, HH pathway plays a key role in determining the fate of sebocytes and is a potential target for the treatment of skin disorders linked to abnormal sebaceous gland function.

#### 2.1.5 Mammary glands

Mammary glands are highly specialized types of sweat glands [75]. There are three important stages of breast development: embryonic stage, pubertal stage and reproductive stage [138]. GLI3, a component of the HH signaling pathway, has recently been described to regulate the formation of bud [42]. After GLI3 is removed, GLI1 expression is inhibited in the mammary gland in the region of placode 3, resulting in the loss or dislocation of bud-pairs 3 and 5. In E11 *Gli*3 xt/xt (extratoes mutant) mice, the absence of WNT signal reporter TOP-Gal in the central region of the mammary gland system suggests that the HH pathway inhibition mediated by GLI3 is necessary prior to early patterning events. Therefore, although HH pathway is active in epidermal appendages (such as HF), it is either inactive or inhibited during the development of mammary glands [86]. It is also interesting that the formation of HF is inhibited, which is conducive to the development of the mammary gland. Recently, it has been found in mice that the blocking of epithelial SHH signal leads to the transformation of some HFs into mammary-gland-like fate [34].

SHH plays an important role in breast cancer. Previous studies have shown that SHH is highly expressed in breast cancer, and the SHH-GLI feedback mechanism contributes to the occurrence and the development of breast cancer [118]. Increased GLI1 expression in breast cancer is associated with aggressive tumor behavior, resulting in higher tumor staging and lymph node status [116]. Compared with patients with low SHH expression level in tumor tissues, patients with high SHH expression level had significantly lower overall survival rate [74]. This conclusion is consistent with previous research by Li's team, that a significant correlation between high HH/SHH expression and adverse prognostic factors exists [116].

#### 2.1.6 Tooth

SHH plays a crucial role in many developmental pathways, including the tooth and nail. During early tooth development in mice, gene expressions including *Ptc*, *Smo*, *Gli1*, *Gli*2, and *Gli*3 are expressed in epithelium and mesenchyme, while SHH is only detected in epithelium, which suggests that SHH is involved in epithelial-stromal and epithelial signals in early tooth development [40]. Tissue reorganization studies have shown that in the early stages of tooth development, the expression of some molecules is indeed regulated by epithelial-mesenchymal interaction, especially in morphogenesis [120].

During tooth development, *Shh* expression has been shown to be localized in epithelial cells [53]. The expression of *Shh* in the early stage of the epithelial thickening stage suggests that the signaling pathway may play a role in the formation of tooth germ and epithelial-mesenchymal interactions. The expression of *Shh* in enamel knot [125] is a signal center in the process of tooth development, which may participate in the formation of cusp by regulating the proliferation of tooth germ. The application of SHH protein in dental beads shows that the downstream gene of the pathway is activated in mesenchymal and the morphology of tooth bud is changed, indicating the role of SHH signal in the epithelial cell proliferation involved in the formation of tooth bud. The results further indicates that although SHH signaling pathway is necessary for the development of normal teeth, there is still functional redundancy between *Gli* genes. Only when *Gli2* and *Gli3* genes are absent can tooth development be mainly affected [40]. In addition to the GLI family, SUFU is also an important factor in regulating the development of mandibular [57].

#### 2.1.7 Nail

Nail structure is consisted of the nail plate, keratin structure without living tissue and four different epithelial tissues: the nail bed, the nail plate, the nail matrix, four distinct epithelial tissues, a keratin structure with no living tissue, the hyponychium and perionychium [41]. The outer surface of each nail is convex, the inner surface is concave, and a part called root is implanted into the groove of the skin. the distal part is called the free edge, and the exposed part is called the body.

SHH plays a significant role in both the formation and development of nails. The appearance of nail from *SHH* ^−/−^ hindlimb is slightly abnormal, which is flatter than the typical wild-type claw in perinatal period, and the middle part is limited. In addition to nails, paw pads also show different phenotypes in *SHH* ^−/−^ [54].

Since a nail normally forms in close proximity to the most distal phalange, the distalmost skeletal element in *SHH* ^−/−^ hind limbs may represent the distal phalanges due to their conical shape and rigid distal end [20].

### 2.2 Other epithelial tissues

#### 2.2.1 Gastric epithelium

Several investigations have evidenced that SHH signaling pathway is also involved in the development of stomach. In *Shh* mutant mice, forestomach area, lined by squamous epithelium, is reduced; while the glandular epithelium of hind stomach is overgrowth [51]. These morphological changes are in accordance with *Shh* expression pattern, as in early stomach development from E11.5 to E15.5, *Shh* is highly expressed in the epithelium of forestomach but lower in the hindstomach [106]. Meanwhile, *Pct1* expressed in mesenchyme show a similar expression pattern of *Shh* [106,125]. When *Shh* expression is disrupted resulting in high expression levels in both forestomach and hind stomach, the glandular epithelium is replaced by squamous epithelium and complex branched pits are replaced by simple unbranched pits [46]. These studies suggest that SHH signaling in stomach may inhibit the formation of glands. Intriguingly, the glandular stomach in *Gli3* rather than *Gli2* knockout mice phenocopies that of *Shh* knockout mice [51], indicating that GLI3 plays a novel activation role in gastric development.

At E18.5 of the later development stage, *Shh* expression expands to the hind stomach, and HH signaling may no longer be exclusive to the mesenchyme, as both PTC1 and GLI1 can also be detected in the epithelial layer [89], which is similar to the adult stomach [9]. SHH derived from parietal cells could promote the mucous compartment expansion. It is worth noting that the postnatal development of stomachs cannot be deciphered for the early lethality of *Shh* knockout mice. The conditional inactivation of HH pathway components in the stomach is awaited to give more precise information [126].

It is now clear that the hedgehog signaling pathway plays a crucial role in gastric homeostasis. Abnormal activation of hedgehog signals is associated with a number of pathological consequences, such as non-atrophic gastritis (NAG) [90], chronic active gastritis (CAG) [72], intestinal metaplasia (IM) [110] and gastric cancer (GC) [1]. According to mortality data provided by the World Health Organization, Gastric Cancer is the fourth most common type of cancer and the second leading cause of cancer deaths worldwide, and is often diagnosed at an advanced stage [28]. SHH signaling pathway is of pivotal importance in cell proliferation, tumor growth and gastric cancer directly [3]. Two major molecules in the HH pathway, SHH and GLI, have been found in GC [139]. Besides, according to Samadani's research, SHH signaling may contribute to the survival of tumor cells, the exact role and mechanism are still unclear [99].

#### 2.2.2 Intestinal epithelium

Early in the development of gut, *Shh* expressed in the endodermal epithelium induces its target gene *Bmp4* expression in the splanchnic mesoderm, which negatively regulates differentiation of both intestinal smooth muscle and neuron formation [65,111]. Meanwhile, the formation of villi, one of the fundamental structures in the small intestinal mucosa, starts at E14.5 in mice when *Shh* expression is downregulated except for the duodenum [11]. In *Shh* mutant mice, villi in duodenum are overgrown and extensively branched, suggesting that SHH seems to restrict the growth of villi [65]. In contrast, IHH stimulates the villi formation as the hypoplastic and reduced number of villi are observed in *Ihh* mutants [89]. Consistent with that, when both SHH and IHH signaling pathways are blocked by *Hhip* overexpression, no villi will form, and the epithelium will remain pseudostratified and poorly differentiated with unfettered proliferation.

After birthing in rodents, crypts are given rise from the intervillous region, where *Shh* is mainly expressed after villi formation. The local diffusion of SHH and IHH within the crypt is thought to inhibit additional crypt formation, thereby guaranteeing orderly crypt spacing, and promoting normal villus development [91]. Moreover, both *Shh* and *Ihh* mutant mice exhibit malrotation of the gut [108], which is probably induced by disrupting the left-right axis formation [115].

SHH, PTC1, and SMO are detected in hyperplastic polyps, adenomas and adenocarcinomas of the colon of both human and mouse [128]. The growth of colon cancer cell lines in vitro could be inhibited by adding cyclopamine (inhibitors of SMO) [5]. Furthermore, abnormal HH pathway activity can indirectly promote colon cancer growth by activating the WNT pathway [129], which is detected active state in 90% of hereditary and sporadic colon cancers [65].

Mechanisms in tumors involves HH pathway can be described as 3 aspects [5]: (1) This pathway is activated by either functionally deficient mutations in inhibitory proteins (such as PTC1, SUFU) or functionally acquired mutations in positive regulators (such as SMO); (2) Overexpression of HH ligands acting in an autocrine/paracrine function to activate the HH pathway, and result in a feedback mitogen that induces tumor growth; (3) Overexpression of HH ligands leads to the regeneration of tumor stem cells.

## 3 Perspective

HH signal is a kind of evolutionarily conserved signaling pathway, which is crucial to cell fate and self-renewal. It has been reported that abnormal HH signal is related to various cancers and other diseases [55], for which scientists have put lots of efforts into studying its function.

In adults, abnormal activation of HH signal is related to congenital disabilities and multiple solid cancers in gastric cancer cell lines, *Shh* is highly expressed, and promotes the transformation of tumor [1]. It is also reported that *Shh* and *Gli* are overexpressed in pancreatic cancer, which suggested that SHH and GLI can be used as prognostic indicators [66]. In addition, the abnormal expression of *Shh* is related to the low survival rate in non-small cell lung cancer (NSCLC). The inhibition of SHH has a significant effect on the invasion and migration of lung cancer cells [48]. Recent clinical results show that inhibiting this pathway has therapeutic benefits. For example, SMO has critical roles in the HH and is a drug target in the treatment of various cancers [102]. Therefore, the basic knowledge of F-GPCRs activation is of great significance not only to reveal the signal transduction mechanism of HH and WNT, but also to develop the therapeutic approaches for clinical application [121,122]. Thus, it is crucial to explore how HH signaling pathway functions in different tissues in order to give insight into the mechanism of HH-related diseases.

Other signal pathways or factors act synergistic roles in HH signaling. It has been reported that GLI could be activated by RAS/AKT, Peptidase Inhibitor 3 (PI3)-Kinase/AKT, rather than HH [93,107]. Although the role of these regulations in development has not been completely unmasked, the interaction and relationship between them remain to be further explored. Biological effects of HH signal may depend on tissue-specific factors and other spatiotemporal regulatory signals, such as epigentic regulation which are unclear. To get new insight into how HH signaling pathway functions in cell fate and stem cell self-renewal, as well as in disease, the mechanism of synergetic regulation of canonical and noncanonical HH signaling pathway, especially what's the molecular switch and how they function, needs to be further investigated.

## Funding

This project is supported by the scientific research start-up funds for specially engaged employees of Sir Run Run Shaw Hospital (Ytp1902), the National Nature Science Foundation of China (31900620), and Open fund for scientific research of Zhe Jiang Chinese Medical University (ZYXZD2019003).

## Conflict of interest

The authors declare that there is no financial or other relationship that might lead to a conflict of the present article. All authors have reviewed the final version of the manuscript and approved it for publication.
